# Biogenesis and Biological Activity of Secondary siRNAs in Plants

**DOI:** 10.1155/2013/783253

**Published:** 2013-02-12

**Authors:** Franck Vazquez, Thomas Hohn

**Affiliations:** Institute of Botany, University of Basel, Zürich-Basel Plant Science Center, Part of the Swiss Plant Science Web, CH-4056 Basel, Switzerland

## Abstract

Two important hallmarks of RNA silencing in plants are (1) its ability to self-amplify by using a mechanism called transitivity and (2) its ability to spread locally and systemically through the entire plant. Crucial advances have been made in recent years in understanding the molecular mechanisms of these phenomena. We review here these recent findings, and we highlight the recently identified endogenous small RNAs that use these advantageous properties to act either as patterning signals in important developmental programs or as a part of regulatory cascades.

## 1. Introduction

RNA silencing is a recently identified mechanism important for the transcriptional and posttranscriptional control of genes and genomes in eukaryotes [1–4]. It also contributes to the defence against viruses [5–10], viroids [11, 12], transposons [13], foreign nucleic acids (e.g., transgenes) [14], and in some cases even against micro-organisms [10, 15, 16].

RNA silencing involves processing of dsRNA by DICERs or DICER-LIKEs to produce small RNA (sRNA) duplexes, capture of the guide siRNA strand by ARGONAUTE (AGO) proteins to form RNA-induced silencing complexes (RISCs) and recognition of homologous target DNA or RNA sequences by RISCs [17–19]. 

**Figure 1:**
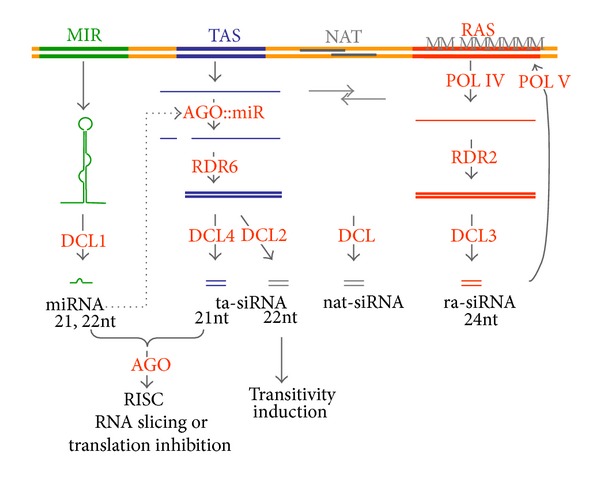
Posttranscriptional and transcriptional gene silencing pathways. Pri-miRNAs consist of a bulged hairpin flanked by unstructured arms. They are transcribed from the relevant MIR genes and are processed predominantly by the DCL1 “Drosha activity” and further by the DCL1 “Dicer activity,” yielding a miRNA duplex. Before processing, the pri-miRNAs, which can be extremely long, are spliced [46]. The DCL1 cofactor, the double-stranded RNA binding protein HYL1, and the 2′-OH ds RNA methyl transferases HEN1, SERRATE, or DAWDLE are not shown [34, 47–51]. Methylation by HEN1serves to protect the miRNA duplex from uridylation and degradation by SDN nucleases [52–57]. With miRNA guide strand and AGO1, a RISC is formed [58, 59], which binds to the cognate target and either slices it or arrests its translation. This step involves the function of cyclophilin 40 and HSP90 [60, 61]. TAS RNAs are transcribed from specific genes too, namely, the TAS genes. TAS RNAs are originally capped and polyadenylated, but they loose the cap and mostly also the poly-A end upon miRNA guided cleavage. They then become processed mainly by DCL4 in a phased way to generate secondary siRNAs, termed ta-siRNAs, which control target mRNAs, similarly as miRNAs [19, 62]. Nat-siRNAs are produced from overlapping dsRNA regions formed by natural antisense transcripts (NATs). Repeat-associated sequences (RASs) give rise to 24nt long ra-siRNAs through the action of DCL3. ra-siRNAs are amplified by POL IV and RDR2 and are involved in DNA and histone methylation by the action of POL V, AGO4, methylases, and chromoproteins [21].

In plants, four endogenous pathways, characterized in *Arabidopsis thaliana* by four specific DICER-LIKE enzymes (DCLs), are involved in sRNA, that is, small interfering (si)RNA and micro (mi)RNA, production [20] (Figure 1). In the RNA-dependent DNA methylation (RdDM) pathway, DCL3 produces 24nt long siRNAs to establish transcriptional gene silencing [21, 22]. This primary RdDM step is further supported by a secondary sRNA-generating machinery that includes RNA polymerases IV and V, AGO4, RNA-dependent RNA polymerase (RDR)2, chromatin remodelling proteins and DNA, and histone methylases [23].

DCL4, together with RDR6 (RNA-dependent RNA polymerase 6), SGS3 (SUPPRESSOR OF GENE SILENCING 3), and DRB4 (dsRNA BINDING PROTEIN 4), produces 21nt long *trans*-acting (ta-) siRNAs from TAS RNA precursors and other types of RNAs [24–28]. DCL2 generates low abundant 22nt long siRNAs from different precursors and mainly acts as a surrogate when DCL4 or DCL3 is mutated or suppressed [29–33]. DCL2 also has a major role in transitivity (discussed in the following) [14]. DCL1 generates 21-22nt miRNAs, and DCL3 generates 23-24nt long-miRNAs from bulged hairpins formed within pri-miRNAs [31, 34–36]. DCL2, DCL3, and DCL4 are also able to generate siRNAs from long hairpins [31, 37]. Silencing by RISC complexes with miRNAs or 21 and 22nt siRNAs can occur by two types of activities. The recognized target RNA is either cleaved (sliced) or its translation is inhibited [38–44]. A clear rule determining one or the other of these alternatives has not yet been identified in each case, although it was suggested in earlier works that slicing is favoured when the complementarity between sRNA and target is perfect or near perfect, while translation inhibition is favoured when the complementarity is imperfect. Because both types of regulations have been reported for a given sRNA/target pair, it is possible that the decision on which mechanism to use depends on specific genetic programming of the different cell types [38, 42, 45].

While DCLs have specific roles in the regulation of genome expression and maintenance, they are all involved in the defence against viruses [8, 17, 64]. For *Cauliflower mosaic Caulimovirus *(CaMV) and *Cabbage leaf curl Begomovirus* (CaLCuV), the genomes which accumulate in the cell nucleus as minichromosomes, all of the four *A. thaliana* DCLs are involved in viral siRNA (vsiRNA) production [8, 30, 65]. In contrast, for RNA viruses, which are restricted to the cytoplasm, 21nt and 22nt siRNAs, produced by DCL4 and DCL2, are mainly involved [29, 65, 66]. The reason for this difference might be that DCL4 and DCL2 can also be available in the cytoplasm, while DCL3 and DCL1 are exclusively localized in the nucleus. Interestingly, viruses have developed countermeasures to meet the silencing strategy, namely, silencing suppressors, which interfere with dicing, RISC-formation, RDR activity, and others [8, 67, 68]. 

The dsRNA substrates used for siRNA production can be formed by annealing of sense and antisense transcripts, by intramolecular pairing leading to formation of hairpins or, in an increasing number of cases, by synthesis from single strand RNA (ssRNA) templates by RDRs. Several host RDRs produce dsRNAs from “aberrant” RNA templates [69]. 

The aberrant nature of these templates is ill defined, but they most likely correspond to uncapped RNAs and/or RNAs without or with only short polyA tails. These aberrant RNAs might arise from premature termination, regulated polyA tail shortening, decapping, or, as discussed in the following, the action of miRNA- or siRNA-guided cleavage of an RNA target.

**Figure 2:**
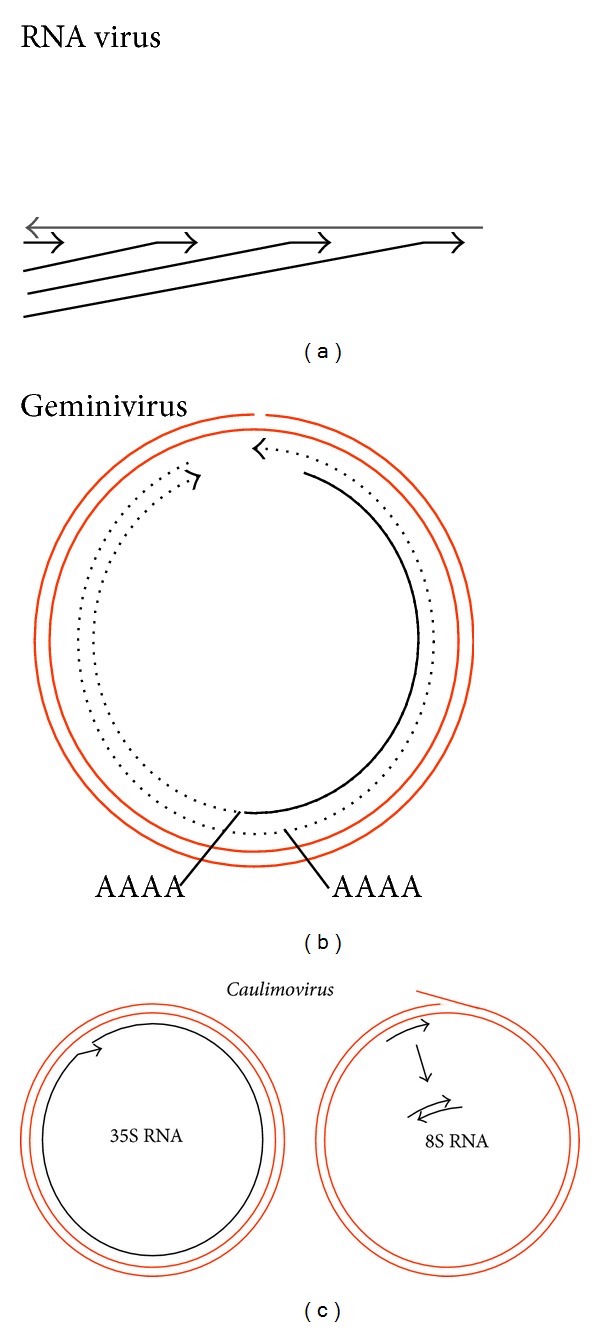
Viral dsRNA formation. (a) RNA-virus RNAs are replicated by viral RNA polymerases producing plus strands from a minus strand and vice versa. Occasionally, dsRNA is formed, namely, before the viral strands can be packaged or are protected by ribosomes. (b) Read-through transcription prior to polyadenylation leads to overlapping transcripts in geminiviruses. (c) *Cauliflower mosaic virus* and other caulimoviruses produce a specific dsRNA covering the leader region. This dsRNA and its siRNA products are thought to act as decoy, the latter by forming nonfunctional RISCs.

Obviously, RNA viruses are replicated by viral RDRs (Figure 2), and despite immediate conversion of the nascent RNA copies into polysomes or virus particles, some might escape and form dsRNAs. Some viruses have dsRNAs as genomes, which are replicated within virus particles and thus are generally shielded from dicing; these particles may occasionally disrupt and release their genome. Geminiviral dsRNAs might arise from bidirectional read-through transcription of the circular DNA genome [64, 70]. In the case of CaMV, a noncoding aberrant “8S” RNA is produced and replicated to dsRNA by the activity of an unknown protein which may correspond to POL II [71].

## 2. A Role of Secondary siRNAs in the Spread of RNA Silencing 

Plants, nematodes, and fungi have the unique property to generate and amplify secondary (sec-) siRNAs. These sec-siRNAs are responsible for the transitivity and spreading of RNA silencing. They can be induced artificially by VIGS vectors carrying host gene sequences or by transgenically expressed genes [14, 72–74]. 

**Figure 3:**
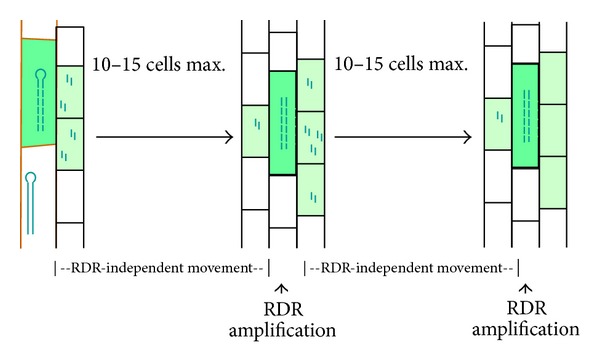
RDR-independent and RDR-dependent spreading of siRNAs. siRNAs can spread about 10 to 15 cell layers. Further spreading requires amplification.

Cell-specific inverted repeat transgenes have been used in *A. thaliana* to trigger the production of siRNAs, which were shown to cause primary posttranscriptional silencing and to spread over 10–15 cells (Figure 3) [75–78]. This short distance movement was shown to be RDR6 independent [78, 79]. Thus, specifically in the case of transgenes, further RNA silencing movement was shown to depend on reiterated RDR-mediated amplification followed by short-distance cell-to-cell movement [78]. In most plants, long-distance RNA silencing spread depends on movement through the phloem. However, long-range root-to-shoot silencing in *Arabidopsis *spreads largely by a series of cell-to-cell short-range mobile silencing events [80]. 

In the case of both, cell-to-cell movement and long-distance movement, the silencing signals include siRNAs [75, 76, 80]. However, whether this movement involves single-stranded siRNAs and/or siRNA duplexes and whether these are bound to dedicated cellular “movement proteins,” like AGOs, remain to be clarified. These sec-siRNAs can be 21nt or 22nt long when generated by DCL4 and DCL2, respectively, and they can be involved in posttranscriptional gene silencing (PTGS), or 24nt long when generated by DCL3, and then be involved in transcriptional gene silencing (TGS).

## 3. Complex Control Mechanisms Guided by sRNAs

**Figure 4:**
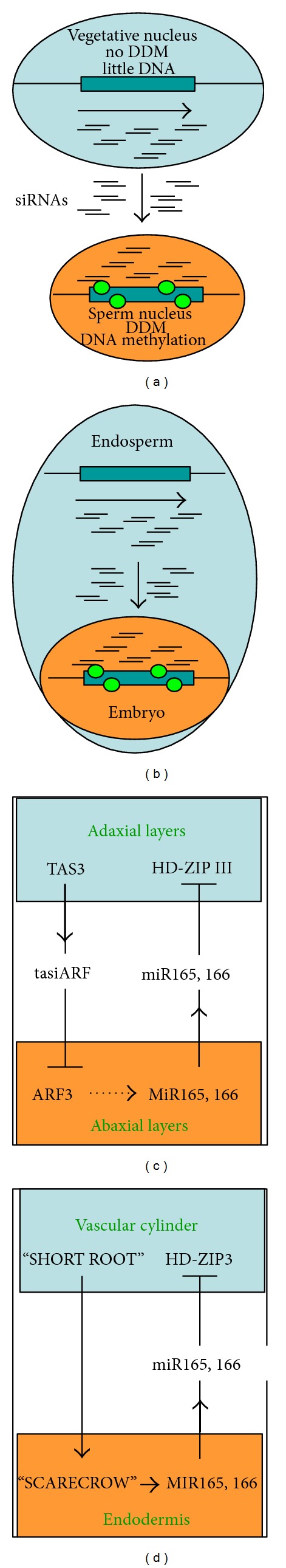
Gene regulation by sRNA movement. (a) Release of siRNAs from transposons in the vegetative nucleus. Those are transported to the sperm nucleus enforcing transposon silencing there. (b) A similar mechanism leads to siRNAs production in the endosperm and their role in embryo chromatin silencing. (c) Control of adaxial and abaxial identity through ta-siRNA and miRNA gradients. (d) Control of xylem patterning through miRNA gradients [63].

siRNA and miRNA transport serves in the plant for various types of complex control mechanisms [81]. Silencing enzymes can be absent or at least underrepresented in certain cells. Thus, the methylase DDM1 is not expressed in the vegetative nucleus of plant embryos. As a consequence, transposons are released, a part of which gives rise to transposon-specific 21nt long siRNAs. Those traffic to sperm cells and there reinforce the silencing of transposons, with the consequence that the embryos are protected from transposition (Figure 4(a)). This mechanism is further reinforced during seed development, where DNA in the endosperm is hypomethylated leading again to transposon release and accumulation of corresponding siRNAs. These move into the embryo to silence transposable elements (Figure 4(b)) [82].

Micro- and ta-si-RNAs can function as morphogens and determine patterning. For example, SHORT ROOT, a transcription factor produced in the vascular cylinder, moves into the endodermis, activates another transcription factor (SCARECROW) there, and together with it activates miR165 and miR166 transcription. These miRNAs move back to the vascular cylinder to encounter their target RNAs, which encode HD-ZIP transcription factors involved in xylem patterning (Figure 4(c)) [63, 99]. 

*Arabidopsis* leaf primordial TAS3a precursor RNA is another example. It is exclusively produced in the L1 and L2 adaxial (upper) leaf layers [81, 100–103]. TAS3 derived ta-siRNAs target auxin response factors (ARFs) 3 and 4 [28, 84, 85, 104–106]. While ARF3 is detected throughout the whole leaf primordial, ARF 4 is exclusively expressed in abaxial (lower) leaf tissue. Since ARFs are targeted throughout the whole leaf primordia, TAS3-derived siRNAs must travel from the adaxial to the abaxial side of the leaf, forming a gradient (Figure 4(d)) and thereby contributing to the establishment of leaf tissue identity. Recent work by Si-Ammour, Windels, and colleagues [92] highlighted a role for siRNAs derived from *TIR/AFB2* auxin receptor (TAAR) transcripts in the regulation of auxin signaling homeostasis and of leaf morphogenesis [91, 92]. However, the movement and the precise role in patterning of siTAARs has not yet been established [91, 92]. 

## 4. A Role of Secondary siRNAs in Transitive RNA Silencing

In some cases, the biogenesis of sec-siRNAs extends towards regions upstream and downstream of the initial target site, a phenomenon called “transitivity” [107–109]. Transitivity in RNA silencing depends on the type and location of the gene affected. For unclear reasons, transgenes are more prone to transitivity than endogenes [74, 108, 110, 111]. A possible explanation might be their high transcription rate that possibly generates more aberrant transcripts and thus more siRNAs than endogens do. Also, transitivity in 5′- to 3′- direction is more often observed than in 3′- to 5′-direction. 

**Figure 5:**
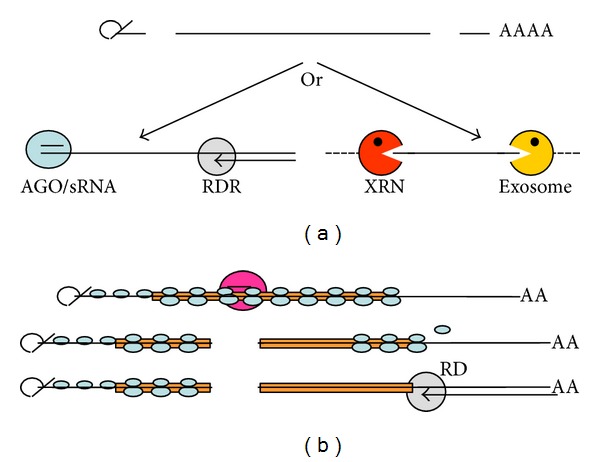
Differential susceptibility of RNAs for transitivity as such and for the direction of transitivity. (a) RDR and RNA-degrading enzymes compete for aberrant RNA. (b) Possible shielding of RNA from RDR activity by scanning and translating ribosomes.

A reason for this differential susceptibility for transitivity might be that the fragments created by RISC-directed cleavage are not only substrates for RDRs but also for exonucleases (e.g., of the XRN family ^1^) and exosomes (Figure 5(a)). One can speculate that RDRs are more efficient or faster enzymes than exonucleases and that the high number of transcripts available for transgenes reaches easily the threshold for triggering of RNA silencing. It is also possible that yet undefined properties of the RNA fragments attract preferentially either the degrading exonucleases or the RDR synthesizing enzymes. As an alternative, the composition of the RISC might determine the fate of the fragment. In fact, this is the case for the programmed production of ta-siRNAs and ra-siRNAs, described in the following.

A reason for the preferred 5′-3′ direction of transitivity might be based on the nature of the RNA fragments created by the initial dicing. We speculate that if the target is within the 5′-UTR or the coding region of an RNA, then the diced 5′-fragment might be shielded from RDR activity by scanning and translating ribosomes, while the 3′-fragment is not. Consequently, only the RNA downstream of the primary dicing site leads to biogenesis of siRNAs (Figure 5(b)). Recent work infecting *Arabidopsis* carrying a GFP transgene with geminivirus VIGS vectors loaded with a series of fragments of this GFP transgene supports this model [111].

## 5. Programmed Triggering of Secondary siRNAs Formation

Genome-wide studies have unravelled several cases of programmed transitivity for endogenes [89]. This programmed transitivity and the corresponding secondary siRNAs originate from various loci and from different types of noncoding (TAS) transcripts (ta-siRNAs) and coding transcripts (sitars, and pha-siRNAs) [24, 25, 85, 89, 91–95].

The common trigger for the biogenesis of sec-siRNAs, ta-siRNAs, and pha-siRNAs is a sRNA-guided slicing event [89, 92, 94, 112]. In the case of certain *trans*-acting siRNAs and some other sec-siRNAs, two slicing events are necessary to trigger their formation from the central released fragment [83, 89]. However this model did not explain the biogenesis of certain low abundant sec-siRNAs that were generated upon a unique slicing event. Bioinformatics analysis comparing sRNA (siRNA and miRNA) target pairs revealed that secondary siRNAs arise predominantly from RNAs that are initially targeted by sRNAs of 22nt in length [113, 114].

Thus, it is now clear that a single slicing event guided by a 22nt sRNA rather than by another size-class sRNA is necessary and sufficient to initiate transitivity. In *A. thaliana*, the genetic observation that *dcl2* mutations eliminate hairpin transgene-induced accumulation of sec-siRNAs, while *dcl4* mutations simply caused a shift in transitive silencing, was an important step towards implicating 22nt in triggering sec-siRNA biogenesis [14]. 

**Figure 6:**
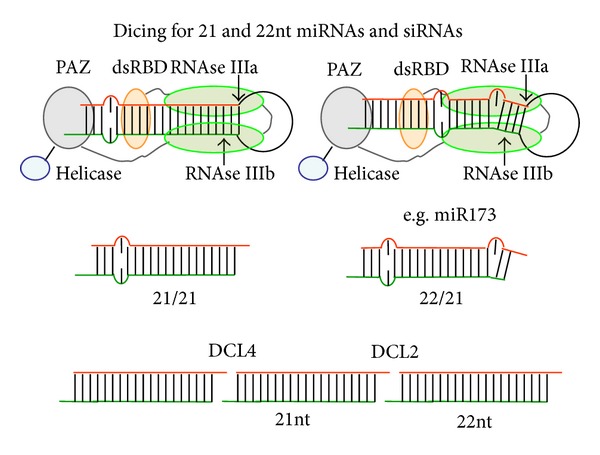
Production of 22nt sRNAs. 22nt miRNAs can be produced from asymmetrically bulged miR-precursors; 22nt siRNAs are produced from dsRNA by DCL2, while DCL4 produces 21nt ones.

22nt long miRNAs are produced from miRNA precursors with an asymmetric hairpin, that is, if bulged on the leading strand, or if a two-nucleotide bulge interrupts the double strand (Figure 6). Mutational analysis showed that removing the bulge in the precursor leads to production of a 21nt rather than a 22nt long miRNA and that although this 21nt miRNA is still active in slicing, it does not initiate transitivity. Likewise, artificial miRNA target pairs led to ta-siRNA production if the miRNA partner was 22nt long and not if it was 21nt long [113–116].

Recent works showed that the presence of a 22nt complementary strand in siRNA duplexes is sufficient to initiate transitivity. Thus, these works showed that the programming of sec-siRNA biogenesis occurs at the level of RISC loading before the sRNA strands are separated. These works suggest that the 22nt sRNA duplexes induce a conformational change in AGO protein that allows them to recruit RDR6, SGS3, or another component of the transitivity machinery [117].

The original discovery of the *trans*-acting pathway highlighted that this class of secondary siRNAs appears to be generated in a phased fashion [24, 25]. This feature, which was confirmed later by the analysis of large sRNA datasets and by molecular genetics experiments, is a hallmark of ta-siRNAs which has been extensively used for the search of novel secondary siRNA loci [85, 90, 106, 118]. 

**Figure 7:**
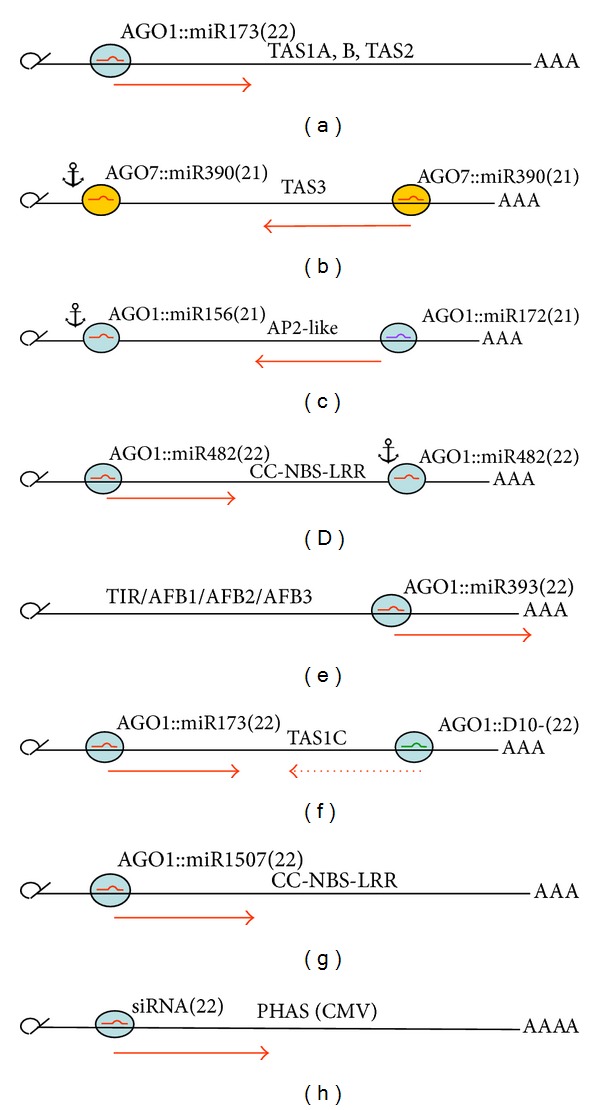
Examples of ta-siRNA and pha-siRNA production. Numbers in parentheses indicate the size of the sRNA considered. the red arrow intends to indicate the direction of transitivity. For details, see the text.

TAS RNAs are capped and polyadenylated and do not code for proteins. Upon slicing and in presence of the cofactor SGS3, TAS RNAs are converted to dsRNAs by RDR6. Upon dicing by DCL4, these dsRNAs spawn swarms of 21- and occasionally 22nt long ta-siRNA duplexes [24, 83, 89, 112, 119]. In *A. thaliana,* four groups of TAS RNAs were found, TAS1, TAS2, TAS3, and TAS4 (Table 1; Figure 7). ta-siRNA production from TAS1a,b,c and TAS2 RNAs is initiated by the 22nt long miR173::AGO1 RISC (e.g., Figures 7(a) and 7(b)) and from TAS4 by the 22nt long miR828::AGO1 RISC [113, 120]. A notable exception concerns TAS3, from which ta-siRNA production is initiated by two 21nt long miR390::AGO7 RISCs, whereby only the second one leads to slicing, while the first one is simply anchored (Figure 7(b)) [83, 105]. 

As miRNAs initiate phased siRNA production from TAS-RNAs, the question is obvious, whether they could also do so from targeted mRNAs. Bioinformatic and molecular biology studies in various plant species showed that in fact they can (Table 1). Many of these cases concern mRNAs encoding pentatricopeptide proteins (PPRs)^2^.

**Figure 8:**
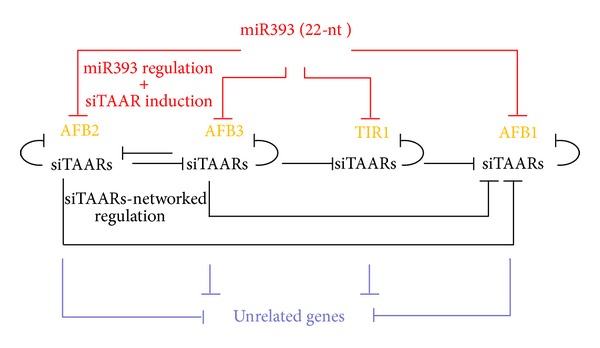
Complex network regulation of siTAARs initiated by miR393. Upon cleavage of TIR1/AFB2 auxin receptor (TAAR) transcripts by miR393 (red lines), secondary siRNAs (siTAARs) are generated. siTAARs regulate the expression of their source transcript in cis (dark lines) of other TAAR transcripts in trans (transverse dark lines) and of unrelated transcripts (blue lines) in trans. The network has important role in auxin homeostasis and plant development.

Recent works by Windels and Vazquez have shown that the regulation of auxin signaling homeostasis depends on a network of sec-siRNAs, termed siTAARs (Figure 8) [91]. Like other sec-siRNAs, siTAARs are generated upon miRNA-guided slicing. In this case, miR393b was shown to guide the cleavage of all four transcripts of the TIR/AFB2 auxin receptor (TAAR) clade and to trigger the biogenesis of sec-siRNAs for at least two of these members. siTAARs were shown to act in *cis* on their own source transcripts as well as in *trans* on homologous *TAAR* transcripts and on unrelated transcripts [92]. siTAARs were shown to be important for some aspect of leaf development, but their precise role in supplementing the function of miR393 remains to be clarified. Analysis of miR393 size in different plant lineages shows that the 22nt size is conserved in at least 7 species out of 22 in which miR393 was sequenced (8) [91]. Thus, if we assume that the biogenesis of siTAARs depends on a 22nt miR393 in other plant species as well, we speculate that the developmental functions of siTAARs might be conserved.

Other recent works showed that also miRNAs that target pathogen resistance R-genes, especially of the nucleotide-binding leucine-rich repeat (NBS-LRR)^3^ type, are controlled by secondary siRNAs that were termed pha-siRNAs. Those were described mainly for Solanaceae [93, 95] and Leguminosae [94]. 

Like TAS RNAs, the corresponding PHAS RNAs are either targeted by double hits, with only one of them leading to slicing while the other is anchored (Table 1; Figures 7(a), 7(b), and 7(d)) or a single hit (Figure 7(h)). In the latter case, usually 22nt miRNAs are involved. In few cases of double hits, two different miRNAs or siRNAs interact with the 5′- and 3′-site [89]. Dicing can occur from the right (Figures 7(b) and 7(c)), the left, or from both sides (Figure 7(f)). 

Recent bioinformatics works have suggested that at least 4 novel TAS families exist in grapevine although they need to be experimentally validated and their biological role remains to be clarified [118].

## 6. Cascades^3^

We discussed previously that the targeting of RNAs by miRNAs and siRNAs can lead to the production of ta-siRNAs, siTAARs, and pha-siRNAs. These secondary sRNAs could initiate further layers of sRNA production and form extensive cascades and networks of gene regulation. Bioinformatics and molecular evidence for this was reported in [90, 113, 121]. 

**Figure 9:**
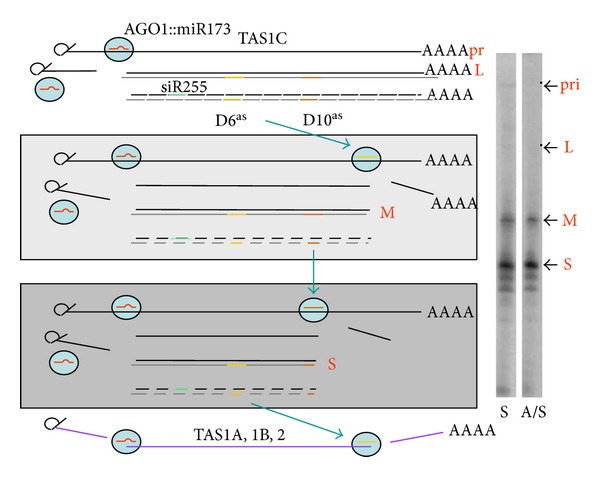
Cascades involving TAS1 and TAS2 processing in *Arabidopsis*. The results rely on the use of CaMV infected plants of which the suppressor TAV interferes with the DCL4/DRB4 activity. The dsRNA intermediates generated by RDR6 accumulating in CaMV infected plants were analyzed. For TAS1c, three main dsRNAs accumulated. The largest and minor one corresponds to a single cut of TAS1c RNA by the AGO1::mir173 RISC, the second one from a double hit by AGO1::miR173 and AGO1::D6^as^, and the third one by AGO1::miR173 and AGO1(2)::D10^as^. D6^as^ and D10^as^ are siRNAs at positions 6 and 10 derived from the antisense strand of the dsRNA intermediate. D10^as^ exists as a 21nt 5′A form likely bound to AGO2 and a 22nt 5′U form bound to AGO1. Notably, D6^as^ RISCs can also target TAS1a, TAS1b, and TAS2 RNAs. On the right, a gel ss showed separating single and double-stranded ta-siRNA precursors (S sense RNAs, AS, antisense RNAs).

Recent works by Rajeswaran et al. [96] have shown that an internal cascade exists for the biogenesis of TAS1 and TAS2 siRNAs in *Arabidopsis* [96]. The work, which takes advantage of the inhibition of DCL4/DRB4 processing step by the CaMV silencing suppressor TAV, allowed to identify the supposed TAS dsRNA intermediates and suggested that the 22nt long siRNA D6(−) produced from TAS1c generates the second hits in TAS1a, TAS1b, and TAS2 RNAs (Figure 9). 

**Figure 10:**
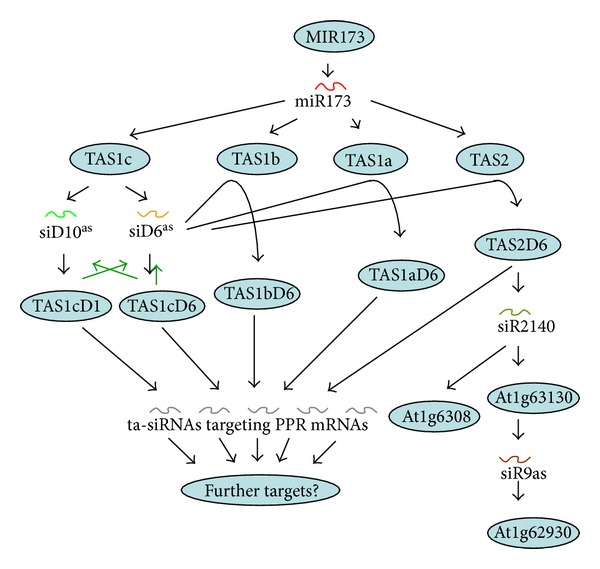
Cascades initiated by miR173 interaction with TAS1 RNAs. Cartouches show precursor RNAs, which give rise to ta-siRNAs and pha-siRNAs that attack further precursor RNAs leading to cascades of gene regulation.

The cascade is known to further continue at least from TAS2 and TAS2D6, giving rise to siR2140 [90], which targets at least two PPR mRNAs. One of these targeted RNAs gives again rise to dsRNA spawning siRNAs, one of which targets a third PPR mRNA (Figure 10). Thus, a cascade originating from miR173 has altogether at least four steps. 

The frequent cases of pha-siRNA production from NB-LRR ^4^ mRNAs of various plant families [93–95] make it likely that at least some of them target other genes. Since there are many NB-LRR-genes present in plants and those related, pha-siRNAs derived from one NB-LRR mRNA could well target a related one. But pha-siRNAs might also target other mRNAs. Shivaprasad et al., for instance, identified a PEN3 like mRNA involved in basal immunity and a proteosome subunit one as secondary targets of tomato miR482 [93].

## Figures and Tables

**Table 1:** MicroRNAs and ta-siRNAs targeting RNAs for secondary siRNA production.

	nt	5′	AGO	Hits	Sec-siRNA source	RNA Targets	Model	References
miR173	22	U	1	1	TAS1a,b,c,2	Pentatricopeptide repeat proteins	A.t.	[24, 25, 83]
miR390	21		7	2	TAS3	Auxin response factors	Plants	[84–86]
miR828	22	U	1	1	TAS4	MYB transcription factors		[87, 88]
miR161/miR400	21		1	2	PPR clade	PPR network	A.t.	[89, 90]
miR393	22		1	1	siTAAR	TAAR network		[89, 91, 92]
miR472	22		1	1	NBS-LRR	NBS-LRR	A.t.	[89]
miR482	22		1	2	NBS-LRR	NBS-LRR	Tomato	[93]
miR780/miR856	21				ATCHX18			[89]
miR2118	22	U	1	1	NBS-LRR; SGS3		Plants	[93, 94]
miR6019	22				NBS-LRR (N)		Tobacco	[95]
miR6020	21				NBS-LRR (N)		Tobacco	[95]
miR2109	22	U	1	1	NBS-LRR		*Medicago*	[94]
miR1507	22	C	1	1	NBS-LRR; DCL2		*Medicago*	[94]
miR1509	22		1	2			*Medicago*	[94]
miR5754	22	U	1	1			*Medicago*	[94]
miR156/miR172	21/21	U/A	1/1	2	AP2-like		*Medicago*	[94]
tas1c D6−	21, 22	U	1, 2, 4		TAS 1a,b,c, TAS2		A.t.	[96]
tas1c D10−	21	A	2		TAS1c		A.t.	[96]
tas1c D10−	22	U	1		TAS1c		A.t.	[96]
tas3 D2−	21				TAS3		Leguminosae	[97]
miR168	22			1	AGO1		A.t., Tomato	[93, 98]

## Endnotes

1. Decapped and deadenylated RNAs are supposedly degraded by XRNs in 5′- to 3′-direction and by the exosomes in 3′- to 5′-direction [109]. Competition between RNA degradation by XRN4 and RDR activity [122, 123] has been observed for several transgenes. However, whether this antagonistic relationship occurs for endogenes is unclear. Indeed, TAS transcripts of the TAS1 and TAS2 families were shown to escape such antagonistic feature [27, 124]. Gregory et al. [124] observed that *xrn4* mutants accumulate low-abundant sRNAs from hundreds of protein-coding loci which were normally not a source for sRNA [124]. These observations showed that XRN4 functions as an antagonist of siRNA formation from certain endogenous transcripts. However, the features determining the different sources of sec-siRNAs remain to be clarified.

2. PPRs are modular superhelical proteins with each of the 35 “pentatrico”-aa-repeats binding to RNA motifs. The genes exist in plant genomes in the hundreds and are responsible for correct chloroplast and mitochondrial gene expression, that is, splicing, processing, editing, translation initiation, and so forth [125].

3. Cascades are also used as means for strict control in other instances. Biological control frequently does not occur in single simple steps but in multiple steps including a cascade of events. This multiplicity allows for precise quantitative control. The single steps can lead either to positive or negative control (activation, repression) [126, 127]. Cascades have been described in mammalian immunology where antibodies are controlled by anti-antibodies and those again by anti-anti-antibodies, and so forth [128], as MAP kinase cascades controlling pathogen response [129] or as transcription factor cascades [130].

4. NB-LRR genes: plants have two main types of defence (R) genes against bacteria, fungi, viruses, and other pathogens: leucine-rich repeat-receptor like kinases (LRR-RLK) and nucleotide-binding leucine-rich repeat (NB-LRR) genes. The former ones are transmembrane proteins recognizing pathogen-associated molecular patterns (PAMPs) outside the cell and involved in the first line of defence alerting the organism. The second class recognizes pathogen effectors inside the cell and induces hypersensitive reactions (HRs) leading to cell death and to systemic acquired resistance (SAR). NBS-LRR genes are abundant and highly variable in all plants analyzed so far [131]. NB-LRR genes seem to evolve fast adapting to the appearance of new variants of pathogens. Due to the high costs of the defence, NB-LRR genes are highly controlled, and recent research revealed a major role in posttranscriptional silencing involving miRNAs and secondary pha-siRNA cascades in this task [94, 95]. Shivaprasad et al. [93] observed that in fact the targeting of NB-LRR RNAs is strongly reduced upon infection by bacteria or viruses. Apparently, effectors and/or silencing suppressors are responsible for this. As a consequence, the plant is alert for a possible pathogen attack due to the presence of NB-LRR-mRNAs but is inhibiting their expression until the pathogen response is really needed [93]. Likewise, mRNAs coding for components of the silencing system are targeted by silencing, for example, SGS3 by miR2118, DCL2 by miR1507, and AGO1 by miR168 (Table 1), and this silencing could be relieved upon virus-directed silencing suppression. Moreover, also the mammalian innate immune system is controlled by anti-inflammation devices [132] and the acquired immune system by networks of anti-antibodies [128].
